# Exploring changes in social spider DNA methylation profiles in all cytosine contexts following infection

**DOI:** 10.1038/s41437-024-00724-y

**Published:** 2024-09-12

**Authors:** David N. Fisher, Jesper Bechsgaard, Trine Bilde

**Affiliations:** 1 School of Biological Sciences, University of Aberdeen, King’s College, Aberdeen, UK; 2 Department of Biology, Section for Genetic Ecology and Evolution, Centre for Ecological Genetics, Aarhus University, Aarhus, Denmark

**Keywords:** DNA methylation, Immunogenetics

## Abstract

Living at high density and with low genetic diversity are factors that should both increase the susceptibility of organisms to disease. Therefore, group living organisms, especially those that are inbred, should be especially vulnerable to infection and therefore have particular strategies to cope with infection. Phenotypic plasticity, underpinned by epigenetic changes, could allow group living organisms to rapidly respond to infection challenges. To explore the potential role of epigenetic modifications in the immune response to a group-living species with low genetic diversity, we compared the genome-wide DNA methylation profiles of five colonies of social spiders (*Stegodyphus dumicola*) in their natural habitat in Namibia at the point just before they succumbed to infection to a point at least six months previously where they were presumably healthier. We found increases in genome- and chromosome-wide methylation levels in the CpG, CHG, and CHH contexts, although the genome-wide changes were not clearly different from zero. These changes were most prominent in the CHG context, especially at a narrow region of chromosome 13, hinting at an as-of-yet unsuspected role of this DNA methylation context in phenotypic plasticity. However, there were few clear patterns of differential methylation at the base level, and genes with a known immune function in spiders had mean methylation changes close to zero. Our results suggest that DNA methylation may change with infection at large genomic scales, but that this type of epigenetic change is not necessarily integral to the immune response of social spiders.

## Introduction

Pathogens and parasites are a key source of mortality and fitness reduction in essentially all organisms. Consequently, pathogens and parasites are expected to be key drivers of evolution, adaptation, and speciation (Van Valen 1973). Parasites and pathogens are expected to play especially key roles in group living species, as disease can spread more easily when organisms live at high densities and frequently interact (Cremer et al. 2007; Nunn et al. 2015). As such, group living organisms are expected to show numerous adaptations to parasites and pathogens (Schmid-Hempel 1988). Variation among individuals in their genes is the raw material upon which selection acts to generate adaptive change and may help protect populations from disease by increasing the chances at least some individuals will be partially resistant (Altermatt and Ebert 2008). However, genetic variation in fitness-relevant traits is expected to be low, and in some group-living organisms individuals may be related due to inbreeding, hence lowering genetic diversity within the group even further (Hatchwell 2010; Settepani et al. 2017). Social groups therefore present a challenge: how do they cope with exposure to parasites and pathogens when living at high densities and when genetic variation among group members is low?

Key candidate mechanisms through which populations with low genetic diversity can still show rapid responses to environmental threats are epigenetic modifications such as histone modification and DNA methylation (Flores et al. 2013). Epigenetic mechanisms can act within an organism’s life to alter gene expression and therefore phenotypes without altering the genetic code (Ledon-Rettig et al. 2012), allowing much more rapid responses to immune challenges compared to adaptive change though changes in allele frequencies across 10 s of generations. For example, in both silkworm (*Bombyx mori*) and a mosquito (*Anopheles albimanus*) the immune response is modulated by DNA methylation in the midgut (Wu et al. 2017; Claudio-Piedras et al. 2020). DNA methylation occurs when the carbon-5 position of a cytosine base gains a methyl group. DNA methylation is one of the more commonly studied epigenetic mechanisms, and in mammals and plants is known to reduce expression of genes when bases within genes promoters are methylated (Bird 2002; He et al. 2011; Elhamamsy 2016). Further, in both plants (Deleris et al. 2016) and mammals (Mostoslavsky and Bergman 1997; Morales-Nebreda et al. 2019) DNA methylation plays a key role in the immune response to both bacterial and viral pathogens. The role of DNA methylation is less clear in arthropods and other invertebrates however, with DNA methylation being associated with both increased, decreased, and stabilised gene expression (Richard et al. 2021; Duncan et al. 2022), and so whether DNA methylation plays an important role in the immune response requires investigating.

Social spiders are an excellent model system to study how group living species resist parasites and pathogens as they live in dense aggregations with constant social interactions among individuals, creating the perfect conditions for disease transmission. The convergent evolution of sociality in spiders is associated with the complete elimination of juvenile dispersal, which leads to an obligatory inbreeding mating system (Lubin and Bilde 2007). Further, social spiders have strong primary female-biased sex ratio, reproductive skew and cooperative brood care. This combination of traits causes reductions in effective population size which leads to very low population genetic diversity (pi < 0.0003), which is a 10x reduction compared to their solitary sister-species (Settepani et al. 2017), and much lower than social insects (Romiguier et al. 2014). Social spiders should therefore be especially susceptible to pathogens. This is supported by the recent findings that social spiders have impaired immune responses compared to their non-social sister-species (Bechsgaard et al. 2022), and that nests do not persist very long, likely due to diseases (Busck et al. 2022). Despite this, populations of social spiders persist.

Comparative genomic approaches have recently shown that genes rapidly evolving in social spiders compared to their solitary sister species include those important in immune function (Tong et al. 2020), indicating this is the ideal taxon to explore how parasites and pathogens drive evolution in group-living organisms. Further, the genome of the social spider *Stegodyphus dumicola* has relatively high methylation level, with a bimodal distribution common in invertebrates, and the patterns are consistent with a role of DNA methylation in local adaptation and also in regulating plasticity in gene expression (Liu et al. 2019; Aagaard et al. 2022, 2024). Genes that are methylated show higher expression (Liu et al. 2019), suggesting that DNA methylation could be a mechanism underpinning changes in gene expression and therefore phenotypic plasticity. Additionally, some genes underpinning expression in plasticity in temperature tolerance phenotypes were generally highly methylated (Aagaard et al. 2024), supporting for a role of DNA methylation in mediating phenotypic plasticity. However, the patterns and functions of DNA methylation in invertebrates and in this taxon are still opaque. The spider immune response involves phagocytosis and encapsulation, production of effector molecules such as hemocyanin and phenoloxidase, and a clotting cascade (as well as constitutive responses; Kuhn-Nentwig and Nentwig 2013; Bechsgaard et al. 2016) all of which could be regulated by gene expression and therefore DNA methylation.

Here we aim to characterise how patterns of DNA methylation change in *S. dumicola* shortly before nest extinction, which is associated with a high bacterial load (Busck et al. 2022). Given the high bacterial load typically present in nests before they die off, we can expect them to be mounting an immune response compared to when they are healthy. By comparing DNA methylation profiles of a nest when it is healthy and when it is about to die off, we can therefore determine the role of DNA methylation in the immune response. We performed this comparison at a range of scales (base, gene, chromosome, whole genome) to gain a holistic understanding of how DNA methylation changes in response to infection.

## Methods

### Sample collection

*Stegodyphus dumicola* is a social spider found in central and southern Africa (Majer et al. 2013). Individuals live in groups of tens to hundreds of individuals. As there is no juvenile or pre-mating dispersal (Lubin and Bilde 2007; Lubin et al. 2009; Smith et al. 2009), social spiders are strictly inbreeding and nest mates are genetically homozygous (Lubin and Bilde 2007; Settepani et al. 2017). New nests are established post-mating by single inseminated females who take off from the natal nest by ballooning (flying on silk threads), which is the only means of long-distance dispersal and lineage propagation (Lubin and Bilde 2007). An existing nest can split by fission to establish a new nest on the same tree or bush, in which case individuals move back and forth between the two nests, but this process is similar to extending the nest rather than propagating the lineage (Lubin and Bilde 2007). Social spiders have a one-year generation time, but a social spider nest has a relatively short lifetime of a few generations due to high predation rates and by succumbing to diseases (Henschel 1998; Crouch and Lubin 2001; Bilde et al. 2007; Busck et al. 2022). Social spiders cooperate in prey capture during which digestive fluids are injected into prey and shared during communal feeding (typically flying insects). Homogenization of bacterial symbionts by social transmission leads to highly similar microbiomes among nest mates (Busck et al. 2020; Rose et al. 2023). Such close interactions also create the ideal conditions for the transmission of pathogens, and indeed it is thought both bacterial and fungal infections are a key source of disease and mortality (Henschel 1998; McEwen et al. 2020; Busck et al. 2022).

**Fig. 1:**
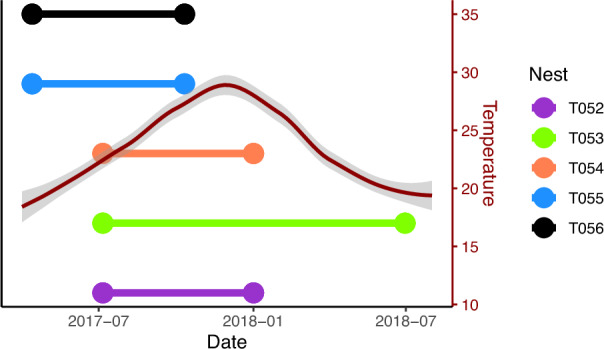
Time points our samples took place, with a smoothed line in red showing the mean temperature (and 95% confidence intervals in grey) during that time to give an idea of season. The earlier time point for each nest is the alive time point, while the latter is the dying time point. Positions of the nests on the y-axis are just for illustration.

As part of a larger study, we located five nests on farmland in northern Namibia (a subset of the nests used in Busck et al. 2022). After locating the first of these nests in April 2017, we returned approximately every three months to locate new nests, record the survivorship of existing nests, and sample three individuals from each nest continuously until the nest “died”. Individuals were extracted by vibrating the capture web or gently squeezing the nest until spiders emerged which were then placed individually in microcentrifuge tubes with ATL buffer (Qiagen, Hilden, Germany) and transferred to a portable freezer. Here we used the three spiders from the last time point a nest was sampled before it “died” (when it was no longer observed as being alive or was observed to be devoid of live spiders) and compared their genome-wide DNA methylation profiles to three spiders from a time point 6–12 months prior to that (hereafter “alive”; see Fig. 1 and Table S1). Once a nest is declining, it shows clear signs of deterioration with less fresh capture silk and less spider activity, and individuals appear in an unhealthy condition and usually die (Trine Bilde and Tharina Bird pers. obs.; Henschel 1998; Crouch and Lubin 2001). Indeed, dead spiders were observed in the dying/or dead nests in this study (Tharina Bird, pers. obs.). Although individuals could in principle leave a dying nest, it is only adult inseminated females that can propagate the lineage and they leave immediately post-mating (Lubin and Bilde 2007; Bilde et al. 2007). Potential movement of remaining spiders from an infected nest at other life stages is however highly limited as it has no propagating function (Lubin et al. 2009; Smith et al. 2009). Importantly, all individuals in the nests were likely infected by disease at the last sampling (Busck et al. 2022). The spiders are synchronous in development, and attempt were made to best possibly match the sizes the sampled spiders (average size in the alive group was 3.4 mm, in the dying group it was 4.1 mm). Given nest extinctions are associated with a large increase in bacterial load both in the last observation before nest death was established (Busck et al. 2022), by comparing infected spiders with those 6–12 months prior of the same nest we aimed to conduct a paired analysis of infected compared to non-infected nests. A paired design is preferable to comparing a group of infected nests and a group of healthy nests as among-colony variation in methylation profiles is large in eusocial hymenoptera (Libbrecht et al. 2016; Marshall et al. 2019; Cardoso-Júnior et al. 2021) and a similar pattern in social spiders would limit our ability to detect a difference between the infected and non-infected groups.

### DNA extraction and sequencing

We extracted DNA from the entire spider using a DNeasy Blood and Tissue kit from Qiagen (Hilden, Germany). We pooled the DNA from the three spiders from each nest at each time point to reduce the impact of individual variation and to assess nest-specific responses. DNA contributions from each spider were equalised by altering the concentrations of each extraction before pooling, see Table S2 for concentrations and scaling factors. We then subjected each of these ten samples to bisulfite treatment. Bisulfite treatment converts unmethylated cytosines to uracils, while methylated cytosines are unconverted (Cokus et al. 2008). Subsequent sequencing and mapping to a reference genome (Ma et al. 2024) then allows one to determine the location of each methylated cytosine. We used DNBSEQ-400 for 100 bp paired-end sequencing (BGI, Hong Kong). To measure the bisulfite conversion error rate we used λDNA, which indicated that >99% of unmethylated cytosines were successfully converted. After sequencing adaptor sequences, contamination, and low-quality reads were removed from the raw data.

### DNA methylation quantification

We identified methylated and unmethylated cytosines using Bismark v 0.23.1 (Krueger and Andrews 2011). First, we indexed the reference genome via bowtie2 with “bismark_genome_preparation”, using the default parameters. Following this, we used the function “bismark” to map the bisulfite converted sequencing reads for a given sample, and the alignment was used to call the methylation status of all cytosines across the genome (default parameters). A small number of alignments can be duplicated, so we removed these using “deduplicate_bismark”. Following this, we extracted the coverage and methylation status for each cytosine base covered by at least one read using “bismark_methylation_extractor”, indicating paired ends (-p), including only a single read from overlapping sections (--no_overlap), and including cytosines in all contexts (--comprehensive). We did this as, while CpG DNA methylation is the most common, both generally and in the *S. dumicola* genome (Liu et al. 2019; note also that the genome has a GC content of 33.3%), important patterns can appear in CHG and CHH contexts; (Dubin et al. 2015; Hämälä et al. 2022). We then created M-bias plots to determine if there were biases in the methylation calls in our reads (Hansen et al. 2012). We observed a bias towards low methylation percentages in the first two bases of the 3’ end, which is often found and results from the sequence of the adapter used. We also observed high variability in the first five bases of the 5’ prime end (Figs. S1–10). As such, we repeated the bismark_methylation_extractor call with --ignore 5 and --ignore_r2 2 to remove these areas of bias. We additionally generated a “bedgraph” format output file containing the position of each cytosine, the coverage, the number of methylated cytosines, the number of unmethylated cytosines, and the percentage of reads that were methylated, for cytosines in each of the CpG, CHG, and CHH contexts. We excluded cytosines from this output file if they had a coverage of less than five reads, as their methylation status would be more uncertain, and those with more than 29 reads, which likely indicated some sort of PCR bias (99.9% of bases had fewer than 30 reads). These steps resulted in thirty output files, three for each of the ten samples.

### DNA methylation analysis

We first determined if the overall percentage of methylated cytosines across the entire genome changed between the alive and dying time points. To do this we followed Liu et al. (2019) in defining each cytosine in our output files as either methylated or not using a binomial test. For each cytosine, the number of methylated reads was entered as the number successes and the coverage as the number of trials, and we tested whether the observed proportion methylated was greater than 0.01 (the error rate estimated with the λDNA, see above). We corrected all *p* values using the Benjamini-Hochberg procedure for multiple tests, and defined bases with a *p* value lower than 0.05 as methylated, and all others as not. For each sample we calculated the genome-wide mean methylation proportion and tested whether there was a clear difference between the alive and dying time points using a Wilcoxon signed rank test. We predicted that overall methylation would increase if methylation was integral to fighting the infection as there would be increased methylation to increase gene expression of relevant pathways. We also calculated the mean methylation proportion per chromosome, and the mean methylation proportion per 1000 bp region (*S. dumicola*’s GC content is 33.3%, and average exon and intron lengths are 381 and 5453 respectively; Liu et al. 2019 which means on average there are 27 CpGs, 22 CHGs, and 115 CHHs per 1000 bp), and used Wilcox-tests to determine if counts in these regions were different between the alive and dying time points and F tests to determine if the variances among these regions were different between the alive and dying time points. We predicted that both the mean and the variance would be greater in the dying group than the alive group, as differentially methylating immune genes would lead to overall-higher but also a more heterogeneous patterns of methylation. Next, we examined the methylation response at the base and gene level using the R package “methylKit” v 1.20.0 (Akalin et al. 2012). For this we returned to the original bedgraph output files. First, we filtered out bases with fewer than 5 or more than 30 reads, but otherwise we retained information on number of reads and percentage that were methylated, rather than assigning a cytosine the status of methylated or not. We also only used bases that were read in at least three samples i.e., their methylation state was observed for at least two different nests.

#### Base-level analyses

We performed an F-test with the McCullagh and Nelder (1989) correction for overdispersion to test for bases that were consistently differentially methylated (either hypo or hypermethylated) between the alive and dying time points, treating each nest as a biological replicate rather than pooling them, including nest identity as a categorical effect, and calculated the mean methylation difference using read coverage as weights. We identified bases that were clearly differentially methylated (DMBs) as those with a change in methylation percentage of at least five percentage points, and with a *p* value, after a Benjamini-Hochberg correction for false discovery rate, of ≤0.05. In the CpG context this corrected *p* value corresponds to an uncorrected *p* value of 1.3086 × 10^−7^, only marginally higher than the commonly-used cut off of 5 × 10^−8^ (recently reviewed by Chen et al. 2021; note that the *S. dumicola* genome is of similar size to the human genome; Risch and Merikangas 1996). We excluded bases on scaffolds 6 and 16 as these are not genuine chromosomes. We then associated the DMBs with an annotated genome using the R packages “plyranges” (Lee et al. 2019) and “Granges” (Lawrence et al. 2013). This allowed us to determine the genome features DMBs tended to be in e.g., regions of coding or repetitive DNA.

#### Gene-level analyses

To test which genes were differentially methylated, we used the original bedgraph output files and summarised the number of methylated and unmethylated cytosines by gene (as defined by the genome annotation file) using the “regionCounts” function in methylKit. We then filtered to remove all genes with more than 100,000 cytosines as these were unusually high (between 95 and 99th percentile and above) and might represent a PCR bias. We then removed all genes that did not feature in at least three samples, as we did for the base-level analysis. We then performed an F-test with the McCullagh and Nelder correction for overdispersion to test for genes that were differentially methylated across samples. This analysis takes into account the total methylated and unmethylated cytosines in each gene (similar to a weighted methylation level; Schultz et al. 2012) when comparing the alive and dying time points. We corrected each *p* value for false discovery rate and reported all those genes with a corrected *p* value of less than 0.05. We performed this for all colonies in the CpG context, but due to the lower rates of methylation we could not do this for the CHH context. In the CHG context, we had to exclude the “alive” sample for nest T54 and the “dying” sample for nest T55 due to no overlap between read cytosines and genes in the annotation file, and so the analysis took place on eight samples, four in each condition.

Finally, to determine if genes with a suggested immune function were differentially methylated between the alive and dying time points, we compiled a list of candidates based on immune genes identified as being present in the congeneric social spider *S. mimosarum* by Bechsgaard et al. (2016) and in the fellow Araneomorphid *Parasteatoda tepidariorum* by both Bechsgaard et al. and Palmer and Jiggins (2015; their Table S5). The FlyBase IDs for these genes (46 in total) are listed in Table S3. We used the program BLAST and the command “tblastx” to identify which of these genes were present in our *S. dumicola* genome. We then used the methylation difference % per gene calculated for the gene-level analysis for these immune genes and tested whether these gene-level scores were different from zero using a two-tailed t-test.

## Results

We obtained on average 249,287,506 alignments per sample, of which on average 2.244% (5,591,062 per alignment) were duplicates and so removed. On average across all ten samples (five nests, both alive and dying) each sample contained 6,851,906,773 cytosines; 1,124,453,155 in the CpG context, 1,244,000,324 in the CHG context, and 4,145,816,481 in the CHH context (some cytosines could not be assigned a context). Mean coverage ranged from 8.808 to 9.975 (Table S1). The mean percentage of methylated cytosines in each was 10.22%, 0.72%, and 0.99% for the CpG, CHG, and CHH contexts respectively (114,911,915, 8,990,689, and 34,680,101 methylated cytosines in the CpG, CHG, and CHH contexts respectively).

### CpG

**Fig. 2:**
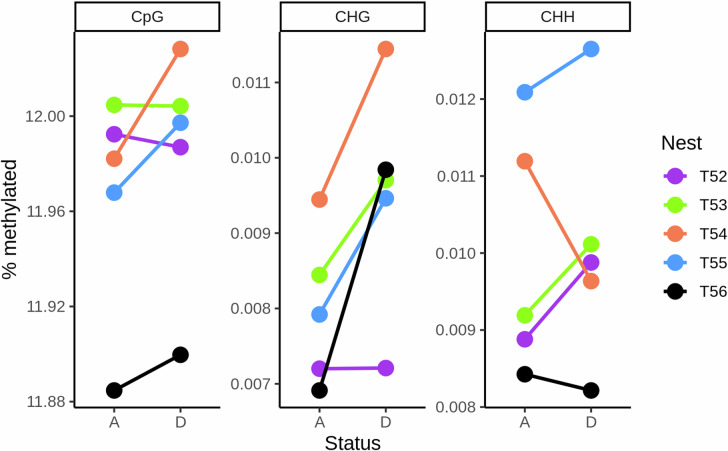
Change in genome-wide methylation percentage of cytosines in each context for nests between being alive and when dying. For each nest (T52-56, depicted with different colours), we show the percentage of the whole genome methylated in each of the three cytosine contexts (CpG, CHG, CHH). In each panel, we give the nests' statuses as either Alive (A), the first time point they were measured at, or Dying (D), the second time point. We link the two statuses for the same nest within one context with a solid line.

Three colonies showed an increase in genome wide methylation rate, while two showed essentially no change (Fig. 2a) meaning there was no clear change overall (paired Wilcoxon signed-rank test, *V* = 3, *p* = 0.313). Chromosomes showed consistent rates of methylation, with increases across all nests when the nest was dying compared to when alive (Fig. S11; paired Wilcoxon signed-rank test, *V* = 240, *p* < 0.001). There was no difference in the variance of methylation rates at the chromosome level between the alive and dying samples (variance alive = 0.0001, variance dying = 0.0001, F-test, *F* = 0.996, df = 69, *p* = 0.988). There was a small increase in mean methylation rates from alive to dying at the level of 1000 base pairs (Wilcoxon signed-rank test, *V* = 1.058 × 10^14^, *p* = 0.001) and a small decrease in the variance in methylation rates (variance alive = 0.1521, variance dying = 0.1517, F-test, *F* = 1.002, df = 14,545,555, *p* < 0.001) but this is a very small difference made clear by the very large sample size. Note the much greater variance in methylation rates at the 1000 base pair level is because many sets of 1000 base pairs have no methylation at all.

**Fig. 3:**
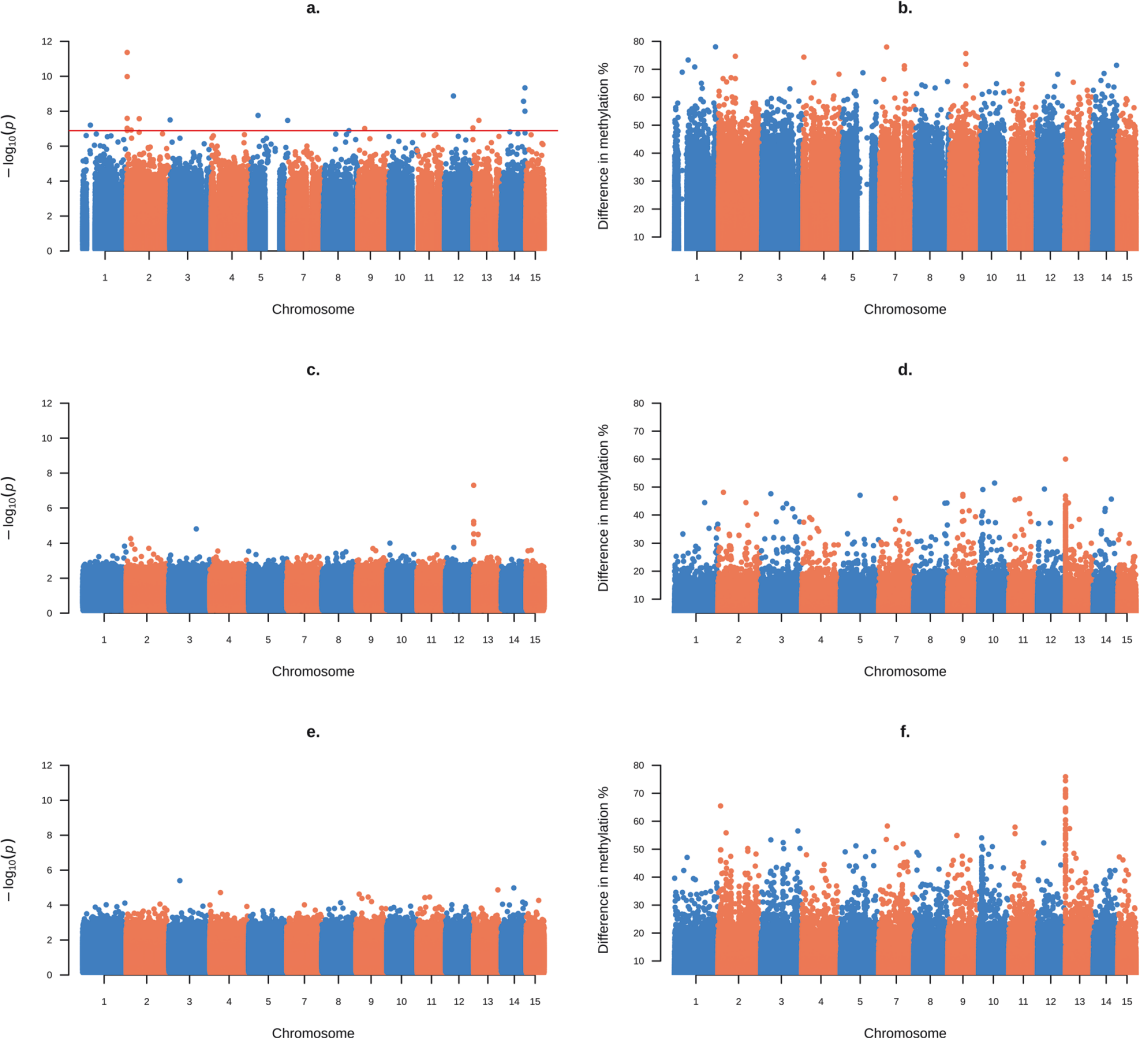
Results of the differential methylation analysis by base for each context. Plot **a**, **b** show the CpG context, **c**, **d** the CHH context and **e**, **f** the CHH context. The left column (**a**, **c**, **e**) show -log10 of *p* values for the statistical test, while the right column (**b**, **d**, **f**) shows the percentage difference in DNA methylation. The red line in **a**. indicates where the *p* value, after a correction for the false discovery rate, was < 0.05 and is our threshold for a clear difference in methylation. Note no bases passed the threshold for statistical significance in the CHG and CHH contexts The use of blue and orange highlights each chromosome.

We identified 19 DMBs (differentially methylated bases) across all nests in the CpG context, eight hypermethylated and 11 hypomethylated (Fig. 3a, b). Of the hypermethylated DMBs, seven were in regions of repetitive DNA, with two LTR retrotransposons (Gypsy, BEL), three LINE retrotransposons (all I-Jockey), one DNA transposons TcMar and two unclassified repeats. The remaining hypermethylated DMB was in a gene intron. Of the hypomethylated DMBs, six were in unclassified repeats in regions of repetitive DNA. The remaining hypomethylated DMBs were in gene introns.

Our analysis of weighted methylation levels per gene revealed a single gene in the that was differentially methylated between alive and dying the in CpG context. This gene had a weighted methylation increase of 3.690 (*q* = 0.016, *p* (fdr) = 0.017), and so was hypermethylated in the dying colonies. Using the NCBI “blastp” tool at https://blast.ncbi.nlm.nih.gov/Blast.cgi we searched the gene’s sequence and found it was 99.59% identical to a heparan-sulfate 6-O-sulfotransferase 2 detected from our study species’ congener *S. mimosarum* (accession number: KFM63315; no other matches were above 88% identical).

The sequences of the 46 putative immune genes we selected were detected 8893 times in the genome. 7417 of these copies appeared in at least three samples for which we could evaluate the methylation change in the CpG context, with a mean methylation difference of 0.259% (min = −8.284, max = 7.925) from alive colonies to dying colonies. The mean is very close to zero, but significantly different due to the large sample size (*t*_7560_ = 22.709, *p* < 0.001, 95% CIs = 0.237 to 0.282).

### CHG

All colonies showed an increase in genome wide methylation rate when dying (Fig. 2b) although this was not clearly different from zero, presumably due to the small sample size (Wilcoxon signed-rank test, *V* = 0, *p* = 0.063). All chromosomes showed increases from alive to dying (Fig. S12; paired Wilcoxon signed rank test, *V* = 444, *p* < 0.001). The variance of methylation rates at the chromosome level was higher in the dying compared to the alive samples (variance alive = 1.511 × 10^−10^, variance dying = 3.618 × 10^−10^, F-test, *F* = 0.417, df = 69, *p* < 0.001). Both the mean and the variance at the level of 1000 base pairs was also higher in the dying compared to alive colonies (Wilcoxon signed rank test, *W* = 1.066 × 10^14^, *p* < 0.001; variance alive = 7.342 × 10^−6^, variance dying = 9.750 × 10^−5^, F-test, *F* = 0.753, df = 14,592,315, *p* < 0.001).

No cytosines were differentially methylated after correcting for multiple testing in the CHG context (Fig. 3c). There was however a clear spike in the *p* values (Fig. 3c) and % differences in methylation (Fig. 3d) at chromosome 13, approximately between bases 704,000 and 821,000. Bases at chromosome 13 were often hypermethylated when dying; the mean methylation percentage difference was +20.872%, compared to +0.125% across the whole genome (see also the increase for chromosome 13 in Fig. S12).

There were no genes that were differentially methylated in the CHG context. For the CHG context there were 7167 copies of immune genes with differential methylation data in at least three samples, with a mean methylation difference of 0.019% (min = −1.645, max = 1.739). Again, the mean is near zero but significantly different due to the large number of genes (*t*_7302_ = 6.498, *p* < 0.001, 95% CIs = 0.013 to 0.025).

### CHH

Three colonies showed an increase in genome wide methylation rate, while two showed a decrease (Fig. 2c). Overall, the change was not different from zero (Wilcoxon signed-rank test, *V* = 6, *p* = 0.813). Chromosomes showed no clear change in median values from alive to dying (Wilcox-test, *V* = 1053, *p* = 0.269; Fig. S13). Variance of methylation rates at the chromosome level was similar between alive and dying colonies (variance alive = 3.092 × 10^−10^, variance dying = 3.673 × 10^−10^, F-test, *F* = 0.842, df = 69, *p* = 0.476). Groups of 1000 bases showed no clear change in median values from alive to dying (Wilcox-test, *V* = 1.073 × 10^14^, *p* = 0.477). Variance at the level of 1000 base pairs was also similar between alive and dying colonies (variance alive = 2.259 × 10^−6^, variance dying = 2.032 × 10^−6^, F-test, *F* = 1.112, df = 14,644,457, *p* < 0.001; note the *p* value is very small due to the large sample size rather than a large effect size).

No cytosines were differentially methylated after correcting for multiple testing in the CHH context (Fig. 3e). There was however a clear spike in the % differences in methylation at the start of chromosome 13, in the same region as for the CHG context (Fig. 3f), but there was no equivalent spike in *p* values (Fig. 3e). Cytosines on chromosome 13 were not consistently hypo- or hypermethylated; the mean methylation difference was −0.104% compared to +0.054% overall (which does not mark it out among the other chromosomes; see Fig. S13).

No gene-level analysis was possible for the CHH context (including for putative immune genes) as we could not calculate weighted methylation levels in this context for each gene, possibly due to the lack of overlap between cytosines methylated in the CHH context and cytosines appearing in gene bodies.

## Discussion

When comparing the genomes of social spiders dying from infection with the genomes of spiders from the same nests while healthy, we saw some increases in the mean and variance of genome- and chromosome-wide methylation levels, but these were only significant in the CHG context. At the per-base level, we saw few differentially methylated-bases in the CpG context, which were mostly associated with regions of repetitive DNA (although this was not an overrepresentation as more than half the genome consists of repeats), and none in the CHG and CHH contexts. There was however a spike of methylation changes in the CHG context in a particular region of chromosome 13. One gene was differentially methylated in the CpG context, but none were in the CHG context and we could not test this in the CHH context. Finally, genes with a known immune function in spiders had cytosines with mean methylation levels close to zero, suggesting this epigenetic modification is not key for changes in gene expression underpinning phenotypic immune responses.

Mean methylation levels in healthy colonies were around 12%, 0.009%, and 0.010% in the CpG, CHG, and CHH contexts respectively, and typically these each increased small amounts when the colonies were dying of infection. However, there was considerable among-nest variation in both means and changes, and so in no context were these differences statistically clear. We had predicted an increase in methylation levels, as we expected genes associated with an immune response to be upregulated and, in this species, methylation in the gene body is associated with higher expression (Liu et al. 2019). At the level of chromosomes and 1000 bases the increases in some of the contexts were clearer, suggesting the lack of clear increase at the nest level may have been due to the low number of colonies (five) rather than by a lack of effect. Similar results have been found in mosquitos *Aedes aegypti*, where infection with Wolbachia was associated with relatively few consistent changes in DNA methylation across the genome (Ye et al. 2013). In contrast, Abudukadier et al. (2021) found that Wolbachia infection in the spider *Hylyphantes graminicola* lead to genome-wide *reductions* in DNA methylation (due to reduced expression of the DNMT1 enzyme, see also Negri et al. 2009). Therefore, whether DNA methylation plays a key role in arthropod plastic immune response remains uncertain. Changes in methylation in response to stress can be increases or decreases depending on whether a gene is highly or lowly methylated (Dixon et al. 2018) and so we may need to investigate more subtle changes than general up or down methylation of genes or bases to determine DNA methylation’s function role.

Research into the role of DNA methylation in phenotypic plasticity has typically focused on methylation in the CpG context, as it is more common than the other contexts. In mammals and plants methylation in the CpG context is clearly associated with changes in gene expression (He et al. 2011; Elhamamsy 2016), but in invertebrates the association between DNA methylation and gene expression is much more variable (Richard et al. 2021; Duncan et al. 2022). Although not statistically significant after correcting for false discovery rate, patterns of differential methylation in the CHG context (and to a lesser extent in the CHH context) were much more localised than in the CpG context, suggesting this context may have a functional role in *S. dumicola*. Intriguingly, recent work in plants has suggested that differential methylation in non-CpG contexts may be associated with environmental variation and response to stress (Li et al. 2020; López et al. 2022; Antro et al. 2022). For example, woodland strawberry (*Fragaria vesca*) show global decrease of 1.8% in CHG methylation in response to salt stress and an increase of 3.1% when challenged with simulated hormone stress, while differential methylation was more common in the CHH context than either of the CpG or CHG contexts (López et al. 2022). While known to play important roles in plants (Kenchanmane Raju et al. 2019), the role of non-CpG context methylation in animals is not yet clear. Non-CpG methylation may play a role in the neural systems of birds and mammals (De Mendoza et al. 2021; Klughammer et al. 2023). More relevantly, non-CpG methylation may be important in insect development, for instance Royle et al. (2024) found increased CHG and CHG methylation for genes in various signalling pathways in the moth *Helicoverpa armigera* in adults compared to pupae, while Araujo and Arias (2021) found non-CpG methylation was key for changes in behaviours between nurse and foraging bees. This importance in development is perhaps down to the role of DNA methylation in controlling alternative splicing, as suggested by work in honey bees *Apis mellifera* (Cingolani et al. 2013) and ants *Camponotus floridanus* and *Harpegnathos saltator* (Bonasio et al. 2012). While these results are intriguing they have not yet confirmed a clear role for CHG and CHH methylation in phenotypic expression, and so along with our results highlight the need for more work to be done on these contexts.

We found mean methylation percentage changes of cytosines in putative immune genes to be around zero, in contrast to our predictions that changes in methylation would be targeted at these genes to alter their expression as part of the immune response. This supports our findings of limited base-level differential methylation and overall suggests limited role of DNA methylation in controlling any plastic responses specifically to infection. In our gene-level analysis we identified one gene that was differentially methylated in the CpG context. Given the number of tests making conclusions about this gene would be unwise. Some studies on invertebrates have found that particular genes are differentially methylated in response to infection, such as genes associated with membrane transport in *A. aegypti* (Ye et al. 2013) and genes associated with the spliceosome, RNA transport, and protein processing in *B. mori* (Huang et al. 2019; see also: Kausar et al. 2022). However, given that methylation is not consistently associated with gene expression in insects (Richard et al. 2021; Duncan et al. 2022), these might be the exceptions rather than the rule.

Although across most of the genome we saw limited changes due to infection, there was a region on chromosome 13 that showed patterns of hypermethylation when infected in both the CHG and CHH contexts (although due to the number of bases tested none of these passed the threshold for statistical significance after correcting for false discovery rate). This was near the start of the chromosome, from approximately bases 704,000 to 821,000. Further analysis showed hypermethylation in this region was similar across genomic features (mean increases of 24.5% in introns and 20.5% in exons) which suggests a non-targeted increase rather directed methylation in order to alter gene expression. We use BLAST to search the NCBI database with the sequences of the two sequences showing the most clear (lowest *p* value) change in methylation (*p* < 3.00 × 10^−5^, compared to > 1.43 × 10^−2^ for all other genes; accession numbers in Table S4). The best match for one sequence was an uncharacterised protein also found in the bark scorpion (*Centruroides sculpturatus*), while the best match for the other was for a fructose aldolase from the bacterium *Mycoplasma anserisalpingitidis*. These hits do not suggest the region of hypermethylation in response to infection we have identified here plays much of a role in the immune system. Given there was also a spike in CHH methylation, an increase in untargeted methylation may have occurred at this region, although why that would be associated with the transition from healthy to infected state is unclear.

In conclusion, we found limited role for specific patterns of DNA methylation in the response to an immune challenge in social spiders *S. dumicola*. Instead, we saw some small genome-wide increases in the mean and variability of each type of methylation. These results suggest that there may be a genome-wide response in DNA methylation to infection, although whether this is adaptive plasticity or a neutral or maladaptive reaction to being infected is not clear. Further work on the prevalence and function of methylation in non-CpG contexts is required to better understand the intriguing patterns of differential methylation in the CHG context at a highly localised site in chromosome 13 we detected.

## Supplementary information

Supplementary materials Tables S1-4, legends of Figures S1–10, Figures S11-13

Supplementary materials Figs S1–10

## Data Availability

The raw sequence data are available through NCBI, BioProject ID: PRJNA1131970. The code files for the generation and analysis of the sequences are available at https://figshare.com/s/8ec0f3ee85a1eb0f2f3e.

## References

[CR1] Aagaard A, Bechsgaard J, Sorenson JG, Sandfeld T, Settepani V, Lund MB et al. (2024) Molecular mechanisms of temperature tolerance plasticity in an arthropod. Genome Biol Evol 16:evae165 39058286 10.1093/gbe/evae165 PMC11979766

[CR2] Aagaard A, Liu S, Tregenza T, Braad Lund M, Schramm A, Verhoeven KJF et al. (2022) Adapting to climate with limited genetic diversity: Nucleotide, DNA methylation and microbiome variation among populations of the social spider *Stegodyphus dumicola*. Mol Ecol 31:5765–5783 36112081 10.1111/mec.16696 PMC9827990

[CR3] Abudukadier A, Huang X, Peng Y, Zhang F, Liu H, Chen J et al. (2021) The potential association between *Wolbachia* infection and DNA methylation in *Hylyphantes graminicola* (Araneae: Linyphiidae). Symbiosis 83:183–191

[CR4] Akalin A, Kormaksson M, Li S, Garrett-Bakelman FE, Figueroa ME, Melnick A et al. (2012) MethylKit: a comprehensive R package for the analysis of genome-wide DNA methylation profiles. Genome Biol 13:87 10.1186/gb-2012-13-10-r87 PMC3491415 23034086

[CR5] Altermatt F, Ebert D (2008) Genetic diversity of Daphnia magna populations enhances resistance to parasites. Ecol Lett 11:918–928 18479453 10.1111/j.1461-0248.2008.01203.x

[CR6] Antro MV, Prelovsek S, Ivanovic S, Gawehns F, Wagemaker NCAM, Mysara M et al. (2023) DNA methylation in clonal Duckweed lineages (*Lemna minor* L.) reflects current and historical environmental exposures. Mol Ecol 32:428–443 10.1111/mec.16757 PMC10100429 36324253

[CR7] Araujo NS, Arias MC (2021) Gene expression and epigenetics reveal species-specific mechanisms acting upon common molecular pathways in the evolution of task division in bees. Sci Rep. 11:3654 33574391 10.1038/s41598-020-75432-8 PMC7878513

[CR8] Bechsgaard J, Jorgensen TH, Jønsson AK, Schou M, Bilde T (2022) Impaired immune function accompanies social evolution in spiders. Biol Lett 18:20220331 36541093 10.1098/rsbl.2022.0331 PMC9768628

[CR9] Bechsgaard J, Vanthournout B, Funch P, Vestbo S, Gibbs RA, Richards S et al. (2016) Comparative genomic study of arachnid immune systems indicates loss of beta-1,3-glucanase-related proteins and the immune deficiency pathway. J Evolut Biol 29:277–291 10.1111/jeb.12780 PMC4744526 26528622

[CR10] Bilde T, Coates KS, Birkhofer K, Bird T, Maklakov AA, Lubin Y et al. (2007) Survival benefits select for group living in a social spider despite reproductive costs. J Evolut Biol 20:2412–2426 10.1111/j.1420-9101.2007.01407.x 17956402

[CR11] Bird A (2002) DNA methylation patterns and epigenetic memory. Genes Dev 16:6–21 11782440 10.1101/gad.947102

[CR12] Bonasio R, Li Q, Lian J, Mutti NS, Jin L, Zhao H et al. (2012) Genome-wide and caste-specific DNA methylomes of the ants *Camponotus floridanus* and *Harpegnathos saltator*. Curr Biol 22:1755–1764 22885060 10.1016/j.cub.2012.07.042 PMC3498763

[CR13] Busck MM, Lund MB, Bird TL, Bechsgaard JS, Bilde T, Schramm A (2022) Temporal and spatial microbiome dynamics across natural populations of the social spider *Stegodyphus dumicola*. FEMS Microbiol Ecol 98:fiac015 10.1093/femsec/fiac015 35147190

[CR14] Busck MM, Settepani V, Bechsgaard J, Lund MB, Bilde T, Schramm A (2020) Microbiomes and specific symbionts of social spiders: compositional patterns in host species, populations, and nests. Front Microbiol 11:1845 32849442 10.3389/fmicb.2020.01845 PMC7412444

[CR15] Cardoso-Júnior CAM, Yagound B, Ronai I, Remnant EJ, Hartfelder K, Oldroyd BP (2021) DNA methylation is not a driver of gene expression reprogramming in young honey bee workers. Mol Ecol 30:4804–4818 34322926 10.1111/mec.16098

[CR16] Chen Z, Boehnke M, Wen X, Mukherjee B (2021) Revisiting the genome-wide significance threshold for common variant GWAS. G3 11:jkaa056 10.1093/g3journal/jkaa056 PMC8022962 33585870

[CR17] Cingolani P, Cao X, Khetani RS, Chen C-C, Coon M, Sammak A et al. (2013) Intronic Non-CG DNA hydroxymethylation and alternative mRNA splicing in honey bees. BMC Genom 14:666 10.1186/1471-2164-14-666 PMC3850688 24079845

[CR18] Claudio-Piedras F, Recio-Tótoro B, Condé R, Hernández-Tablas JM, Hurtado-Sil G, Lanz-Mendoza H (2020) DNA Methylation in *Anopheles albimanus* modulates the midgut immune response against plasmodium berghei. Front Immunol 10:3025 31993053 10.3389/fimmu.2019.03025 PMC6970940

[CR19] Cokus SJ, Feng S, Zhang X, Chen Z, Merriman B, Haudenschild CD et al. (2008) Shotgun bisulphite sequencing of the *Arabidopsis* genome reveals DNA methylation patterning. Nature 452:215–219 18278030 10.1038/nature06745 PMC2377394

[CR20] Cremer S, Armitage SAO, Schmid-Hempel P (2007) Social immunity. Curr Biol 17:R693–R702 17714663 10.1016/j.cub.2007.06.008

[CR21] Crouch T, Lubin Y (2001) Population stability and extinction in a social spider *Stegodyphus mimosarum* (Araneae: Eresidae). Biol J Linn Soc 72:409–417

[CR22] De Mendoza A, Poppe D, Buckberry S, Pflueger J, Albertin CB, Daish T et al. (2021) The emergence of the brain non-CpG methylation system in vertebrates. Nat Ecol Evol 5:369–378 33462491 10.1038/s41559-020-01371-2 PMC7116863

[CR23] Deleris A, Halter T, Navarro L (2016) DNA methylation and demethylation in plant immunity. Annu Rev Phytopathol 54:579–603 27491436 10.1146/annurev-phyto-080615-100308

[CR24] Dixon G, Liao Y, Bay LK, Matz MV (2018) Role of gene body methylation in acclimatization and adaptation in a basal metazoan. Proc Natl Acad Sci 115:13342–13346 30530646 10.1073/pnas.1813749115 PMC6310852

[CR25] Dubin MJ, Zhang P, Meng D, Remigereau MS, Osborne EJ, Casale FP et al. (2015) DNA methylation in *Arabidopsis* has a genetic basis and shows evidence of local adaptation. eLife 4:e05255 10.7554/eLife.05255 PMC4413256 25939354

[CR26] Duncan EJ, Cunningham CB, Dearden PK (2022) Phenotypic plasticity: what has DNA methylation got to do with it? Insects 13,110 10.3390/insects13020110 PMC8878681 35206684

[CR27] Elhamamsy AR (2016) DNA methylation dynamics in plants and mammals: overview of regulation and dysregulation. Cell Biochem Funct 34:289–298 27003927 10.1002/cbf.3183

[CR28] Flores KB, Wolschin F, Amdam GV (2013) The role of methylation of DNA in environmental adaptation. Integr Comp Biol 53:359–372 23620251 10.1093/icb/ict019 PMC3710460

[CR29] Hämälä T, Ning W, Kuittinen H, Aryamanesh N, Savolainen O (2022) Environmental response in gene expression and DNA methylation reveals factors influencing the adaptive potential of Arabidopsis lyrata. eLife 11:e83115 36306157 10.7554/eLife.83115 PMC9616567

[CR30] Hansen KD, Langmead B, Irizarry RA (2012) BSmooth: from whole genome bisulfite sequencing reads to differentially methylated regions. Genome Biol 13:1–10 10.1186/gb-2012-13-10-r83 PMC3491411 23034175

[CR31] Hatchwell BJ (2010) Cryptic kin selection: Kin structure in vertebrate populations and opportunities for kin-directed cooperation. Ethology 116:203–216

[CR32] He XJ, Chen T, Zhu JK (2011) Regulation and function of DNA methylation in plants and animals. Cell Res 21:442–465 21321601 10.1038/cr.2011.23 PMC3152208

[CR33] Henschel JR (1998) Predation on social and solitary individuals of the spider *Stegodyphus dumicola* (Araneae, Eresidae). J Arachnol 26:61–69

[CR34] Huang H, Wu P, Zhang S, Shang Q, Yin H, Hou Q et al. (2019) DNA methylomes and transcriptomes analysis reveal implication of host DNA methylation machinery in BmNPV proliferation in *Bombyx mori*. BMC Genom 20:736 10.1186/s12864-019-6146-7 PMC6792228 31615392

[CR35] Kausar S, Liu R, Gul I, Abbas MN, Cui H (2022) Transcriptome sequencing highlights the regulatory role of DNA methylation in immune-related genes’ expression of chinese oak silkworm, *Antheraea pernyi*. Insects 13:296 35323594 10.3390/insects13030296 PMC8951095

[CR36] Kenchanmane Raju SK, Ritter EJ, Niederhuth CE (2019) Establishment, maintenance, and biological roles of non-CG methylation in plants. Essays Biochem 63:743–755 31652316 10.1042/EBC20190032 PMC6923318

[CR37] Klughammer J, Romanovskaia D, Nemc A, Posautz A, Seid CA, Schuster LC et al. (2023) Comparative analysis of genome-scale, base-resolution DNA methylation profiles across 580 animal species. Nat Commun 14:232 36646694 10.1038/s41467-022-34828-y PMC9842680

[CR38] Krueger F, Andrews SR (2011) Bismark: a flexible aligner and methylation caller for Bisulfite-Seq applications. Bioinformatics 27:1571–1572 21493656 10.1093/bioinformatics/btr167 PMC3102221

[CR39] Kuhn-Nentwig L, Nentwig W (2013) The immune system of spiders. In: Spider Ecophysiology, Springer-Verlag Berlin Heidelberg, p 81–91

[CR40] Lawrence M, Huber W, Pagès H, Aboyoun P, Carlson M, Gentleman R et al. (2013) Software for computing and annotating genomic ranges. PLOS Comput Biol 9:e1003118 23950696 10.1371/journal.pcbi.1003118 PMC3738458

[CR41] Ledon-Rettig CC, Richards CL, Martin LB (2012) Epigenetics for behavioral ecologists. Behav Ecol 24:311–324

[CR42] Lee S, Cook D, Lawrence M (2019) Plyranges: a grammar of genomic data transformation. Genome Biol 20:1–10 30609939 10.1186/s13059-018-1597-8 PMC6320618

[CR43] Li R, Hu F, Li B, Zhang Y, Chen M, Fan T et al. (2020) Whole genome bisulfite sequencing methylome analysis of mulberry (*Morus alba*) reveals epigenome modifications in response to drought stress. Sci Rep. 10:8013 32415195 10.1038/s41598-020-64975-5 PMC7228953

[CR44] Libbrecht R, Oxley PR, Keller L, Kronauer DJC (2016) Robust DNA methylation in the clonal raider ant brain. Curr Biol 26:391–395 26804553 10.1016/j.cub.2015.12.040 PMC5067136

[CR45] Liu S, Aagaard A, Bechsgaard J, Bilde T (2019) DNA methylation patterns in the social spider, Stegodyphus dumicola. Genes 10:137 30759892 10.3390/genes10020137 PMC6409797

[CR46] López M-E, Roquis D, Becker C, Denoyes B, Bucher E (2022) DNA methylation dynamics during stress response in woodland strawberry (Fragaria vesca). Hortic Res 9:uhac174 36204205 10.1093/hr/uhac174 PMC9533225

[CR47] Lubin Y, Bilde T (2007) The evolution of sociality in spiders. Adv Study Behav 37:83–145

[CR48] Lubin Y, Birkhofer K, Berger-Tal R, Bilde T (2009) Limited male dispersal in a social spider with extreme inbreeding. Biol J Linn Soc 97:227–234

[CR49] Ma J, Bechsgaard J, Aagaard A, Villesen P, Bilde T, Schierup M (2024) Sociality in spiders is an evolutionary dead-end. https://www.biorxiv.org/content/10.1101/2024.04.22.590577v1 10.1101/gr.279503.124 PMC11960701 39978820

[CR50] Majer M, Svenning JC, Bilde T (2013) Habitat productivity constrains the distribution of social spiders across continents - case study of the genus *Stegodyphus*. Front Zool 10:1–10 23433065 10.1186/1742-9994-10-9 PMC3599804

[CR51] Marshall H, Lonsdale ZN, Mallon EB (2019) Methylation and gene expression differences between reproductive and sterile bumblebee workers. Evol Lett 3:485–499 31636941 10.1002/evl3.129 PMC6791180

[CR52] McCullagh P, Nelder JA (1989) Generalized Linear Models. Chapman & Hall/CRC, New York

[CR53] McEwen BL, Lichtenstein JLL, Fisher DN, Wright CM, Chism GT, Pinter-Wollman N, et al. (2020) Predictors of colony extinction vary by habitat type in social spiders. Behav Ecol Sociobiol 74:1–9 10.1007/s00265-019-2781-x PMC7236762 32431472

[CR54] Morales-Nebreda L, McLafferty FS, Singer BD (2019) DNA methylation as a transcriptional regulator of the immune system. Transl Res 204:1–18 30170004 10.1016/j.trsl.2018.08.001 PMC6331288

[CR55] Mostoslavsky R, Bergman Y (1997) DNA methylation: regulation of gene expression and role in the immune system. Biochim et Biophys Acta 1333:1997–2026 10.1016/s0304-419x(97)00010-3 9294017

[CR56] Negri I, Franchini A, Gonella E, Daffonchio D, Mazzoglio PJ, Mandrioli M et al. (2009) Unravelling the *Wolbachia* evolutionary role: the reprogramming of the host genomic imprinting. Proc R Soc B 276:2485–2491 19364731 10.1098/rspb.2009.0324 PMC2690474

[CR57] Nunn CL, Jordán F, McCabe CM, Verdolin JL, Fewell JH (2015) Infectious disease and group size: more than just a numbers game. Philos Trans R Soc Lond Ser B, Biol Sci 370:20140111 25870397 10.1098/rstb.2014.0111 PMC4410377

[CR58] Palmer WJ, Jiggins FM (2015) Comparative genomics reveals the origins and diversity of arthropod immune systems. Mol Biol Evol 32:2111–2129 25908671 10.1093/molbev/msv093 PMC4833078

[CR59] Richard G, Jaquiéry J, Le Trionnaire G (2021) Contribution of epigenetic mechanisms in the regulation of environmentally-induced polyphenism in insects. Insects 12:649 34357309 10.3390/insects12070649 PMC8304038

[CR60] Risch N, Merikangas K (1996) The future of genetic studies of complex human diseases. Science 273:1516–1517 8801636 10.1126/science.273.5281.1516

[CR61] Romiguier J, Lourenco J, Gayral P, Faivre N, Weinert LA, Ravel S et al. (2014) Population genomics of eusocial insects: the costs of a vertebrate-like effective population size. J Evolut Biol 27:593–603 10.1111/jeb.12331 26227898

[CR62] Rose C, Lund MB, Søgård AM, Busck MM, Bechsgaard JS, Schramm A et al. (2023) Social transmission of bacterial symbionts homogenizes the microbiome within and across generations of group-living spiders. ISME Commun 3:60 37330540 10.1038/s43705-023-00256-2 PMC10276852

[CR63] Royle JW, Hurwood D, Sadowski P, Dudley KJ (2024) Non-CG DNA methylation marks the transition from pupa to adult in *Helicoverpa armigera.* Insect Mol Biol 33:493–502 38668923 10.1111/imb.12917

[CR64] Schmid-Hempel P (1988) Parasites in Social Insects. In: Schmid-Hempel P, (eds.), Princeton University Press: Princeton.

[CR65] Schultz MD, Schmitz RJ, Ecker JR (2012) Leveling’ the playing field for analyses of single-base resolution DNA methylomes. Trends Genet 28:583–585 23131467 10.1016/j.tig.2012.10.012 PMC3523709

[CR66] Settepani V, Schou MF, Greve M, Grinsted L, Bechsgaard J, Bilde T (2017) Evolution of sociality in spiders leads to depleted genomic diversity at both population and species levels. Mol Ecol 26:4197–4210 28570031 10.1111/mec.14196

[CR67] Smith D, Van Rijn S, Henschel J, Bilde T, Lubin Y (2009) Amplified fragment length polymorphism fingerprints support limited gene flow among social spider populations. Biol J Linn Soc 97:235–246

[CR68] Tong C, Najm GM, Pinter-Wollman N, Pruitt JN, Linksvayer TA, Pisani D (2020) Comparative genomics identifies putative signatures of sociality in spiders. Genome Biol Evol 12:122–133 31960912 10.1093/gbe/evaa007 PMC7108510

[CR69] Van Valen L (1973) A new evolutionary law. Evolut Theory 1:1–30

[CR70] Wu P, Jie W, Shang Q, Annan E, Jiang X, Hou C et al. (2017) DNA methylation in silkworm genome may provide insights into epigenetic regulation of response to *Bombyx mori* cypovirus infection. Sci Rep 7:16013 10.1038/s41598-017-16357-7 PMC5700172 29167521

[CR71] Ye YH, Woolfit M, Huttley GA, Rancès E, Caragata EP, Popovici J et al. (2013) Infection with a virulent strain of *Wolbachia* disrupts genome wide-patterns of cytosine methylation in the mosquito *Aedes aegypti* (K Bourtzis, Ed.). PLoS ONE 8:e66482 23840485 10.1371/journal.pone.0066482 PMC3686743

