# Supplementary figures and images for: Exploring changes in social spider DNA methylation profiles in all cytosine contexts following infection
Source: Heredity (Edinb). 2024 Sep 12;133(6):410–7. doi: 10.1038/s41437-024-00724-y (PMC11589119; doi:10.1038/s41437-024-00724-y)

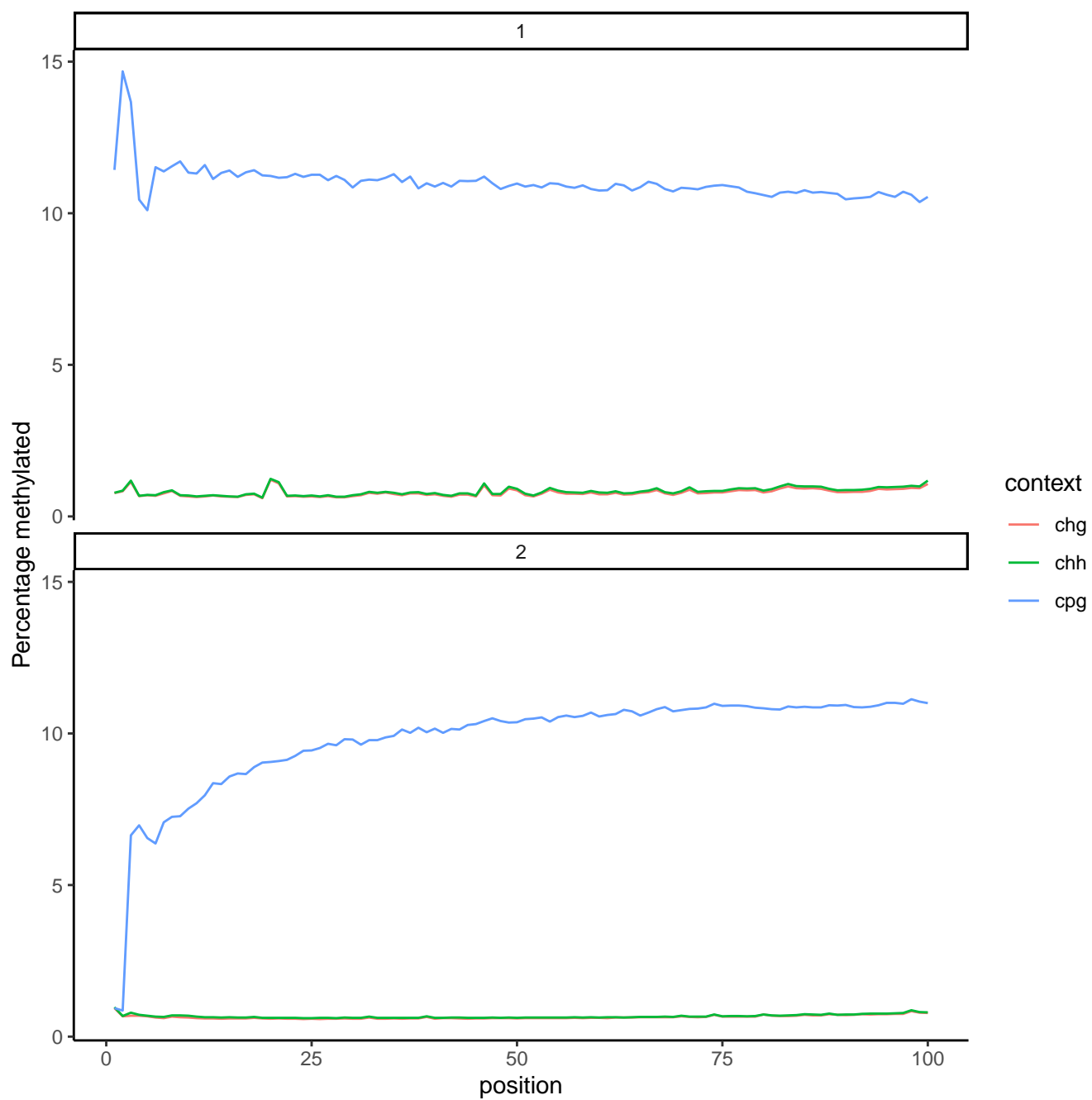

Supplement: Supplementary file 2 — Supplementary materials Figs S1–10 [file 41437_2024_724_MOESM2_ESM.zip › Spider Meth Figs S1-10 m-bias plots/Spider Meth Fig S1 T52 Alive.pdf]

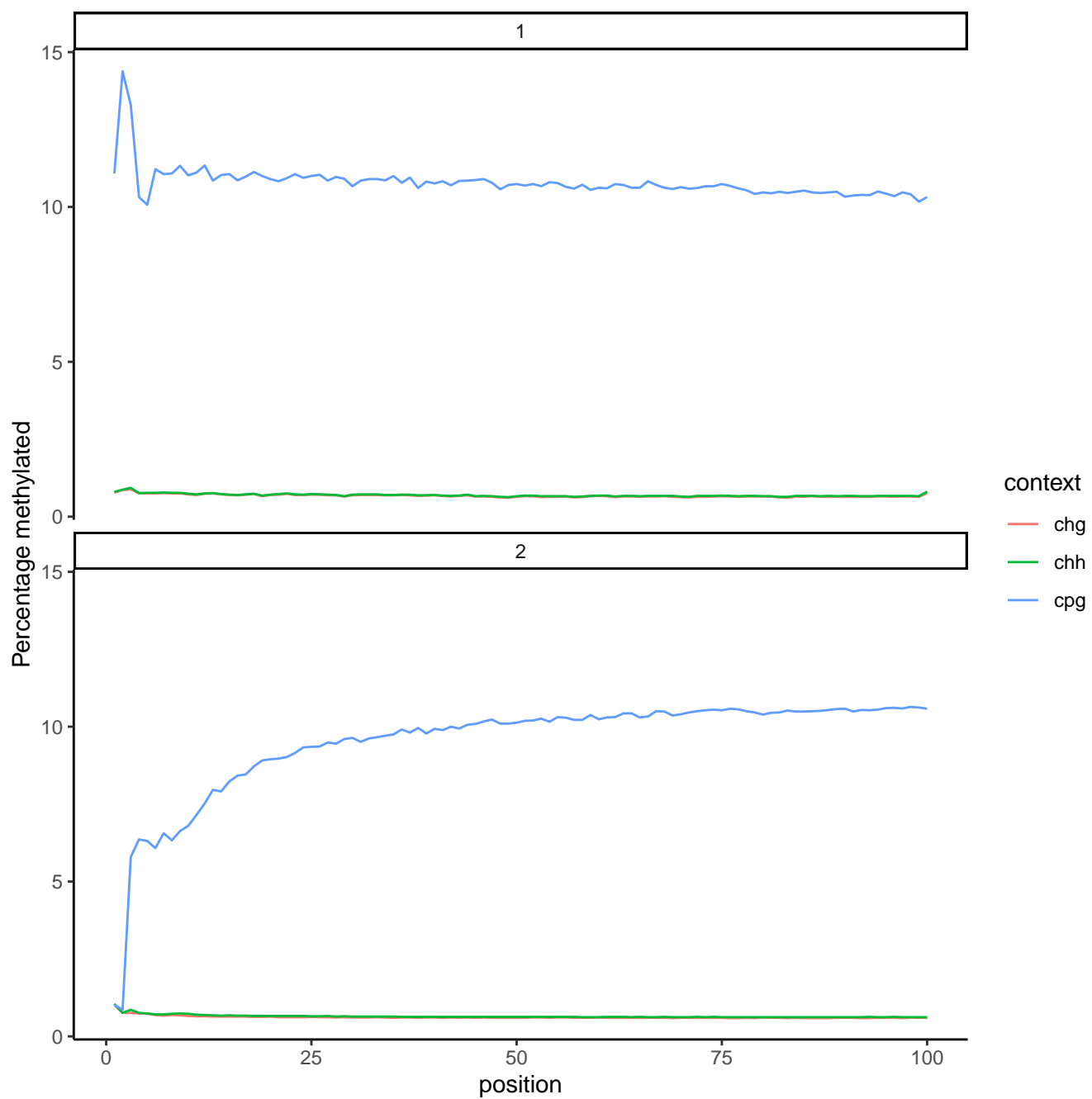

Supplement: Supplementary file 2 — Supplementary materials Figs S1–10 [file 41437_2024_724_MOESM2_ESM.zip › Spider Meth Figs S1-10 m-bias plots/Spider Meth Fig S10 T56 Dying.pdf]

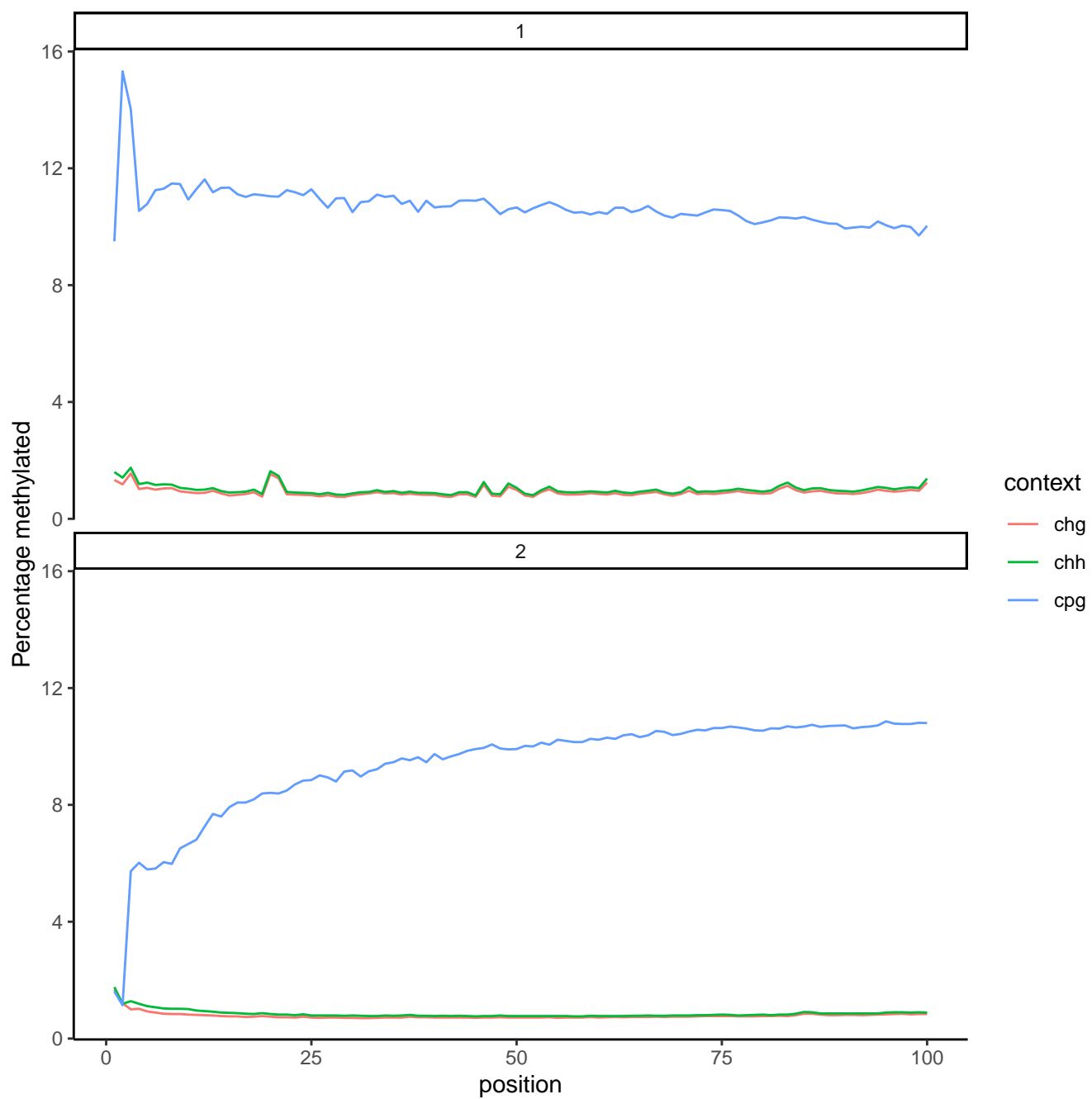

Supplement: Supplementary file 2 — Supplementary materials Figs S1–10 [file 41437_2024_724_MOESM2_ESM.zip › Spider Meth Figs S1-10 m-bias plots/Spider Meth Fig S2 T52 Dying.pdf]

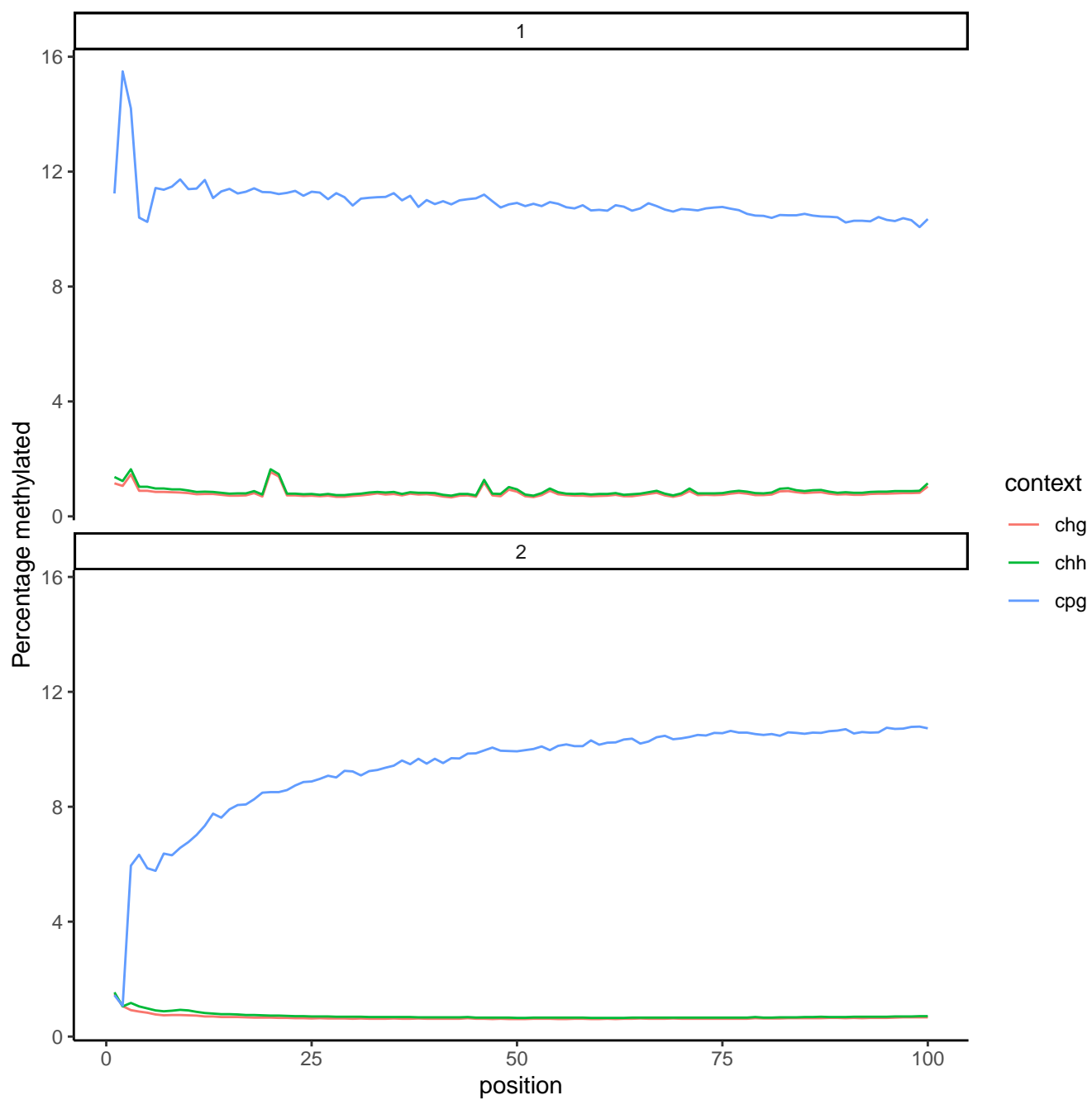

Supplement: Supplementary file 2 — Supplementary materials Figs S1–10 [file 41437_2024_724_MOESM2_ESM.zip › Spider Meth Figs S1-10 m-bias plots/Spider Meth Fig S3 T53 Alive.pdf]

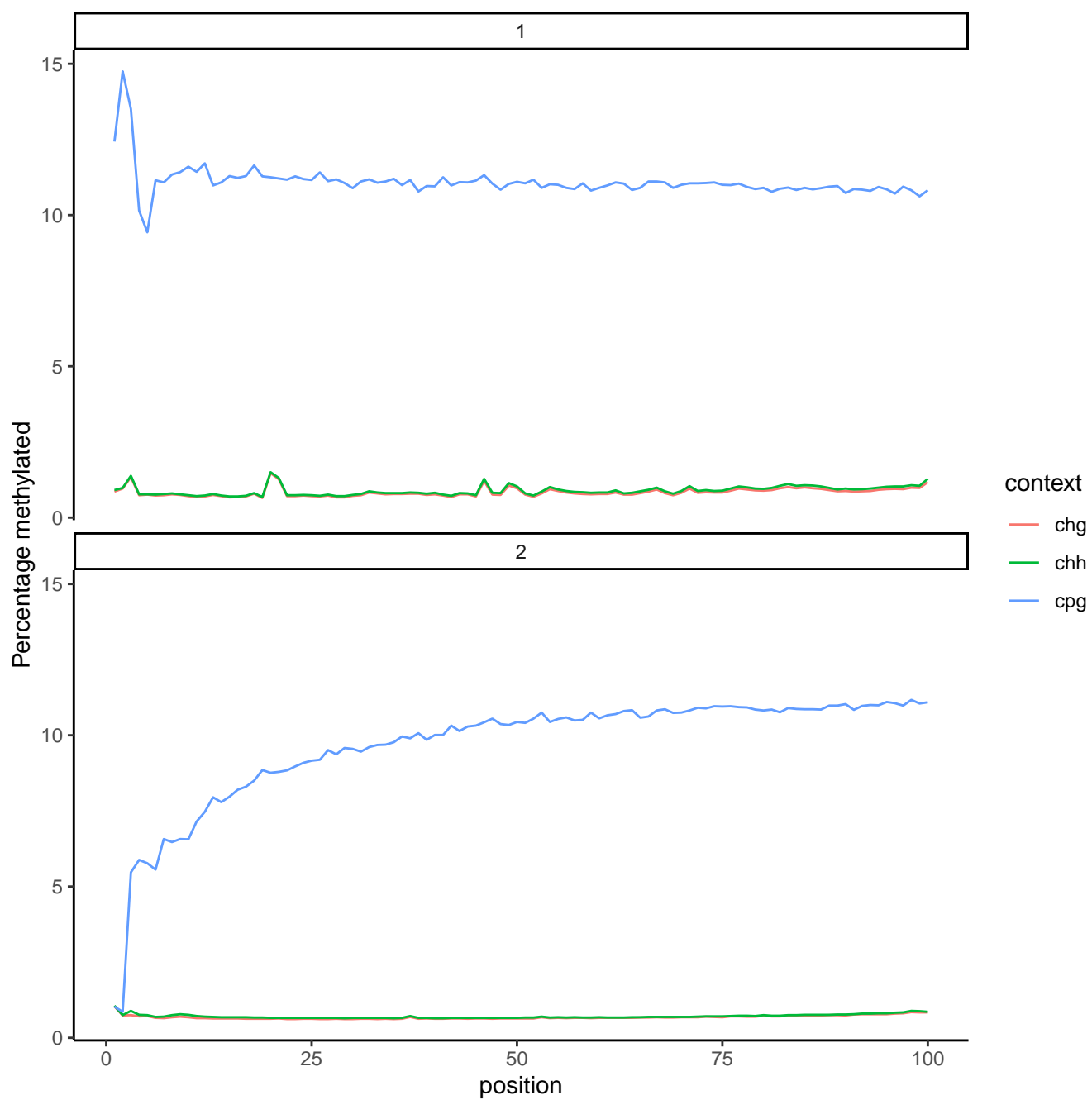

Supplement: Supplementary file 2 — Supplementary materials Figs S1–10 [file 41437_2024_724_MOESM2_ESM.zip › Spider Meth Figs S1-10 m-bias plots/Spider Meth Fig S4 T53 Dying.pdf]

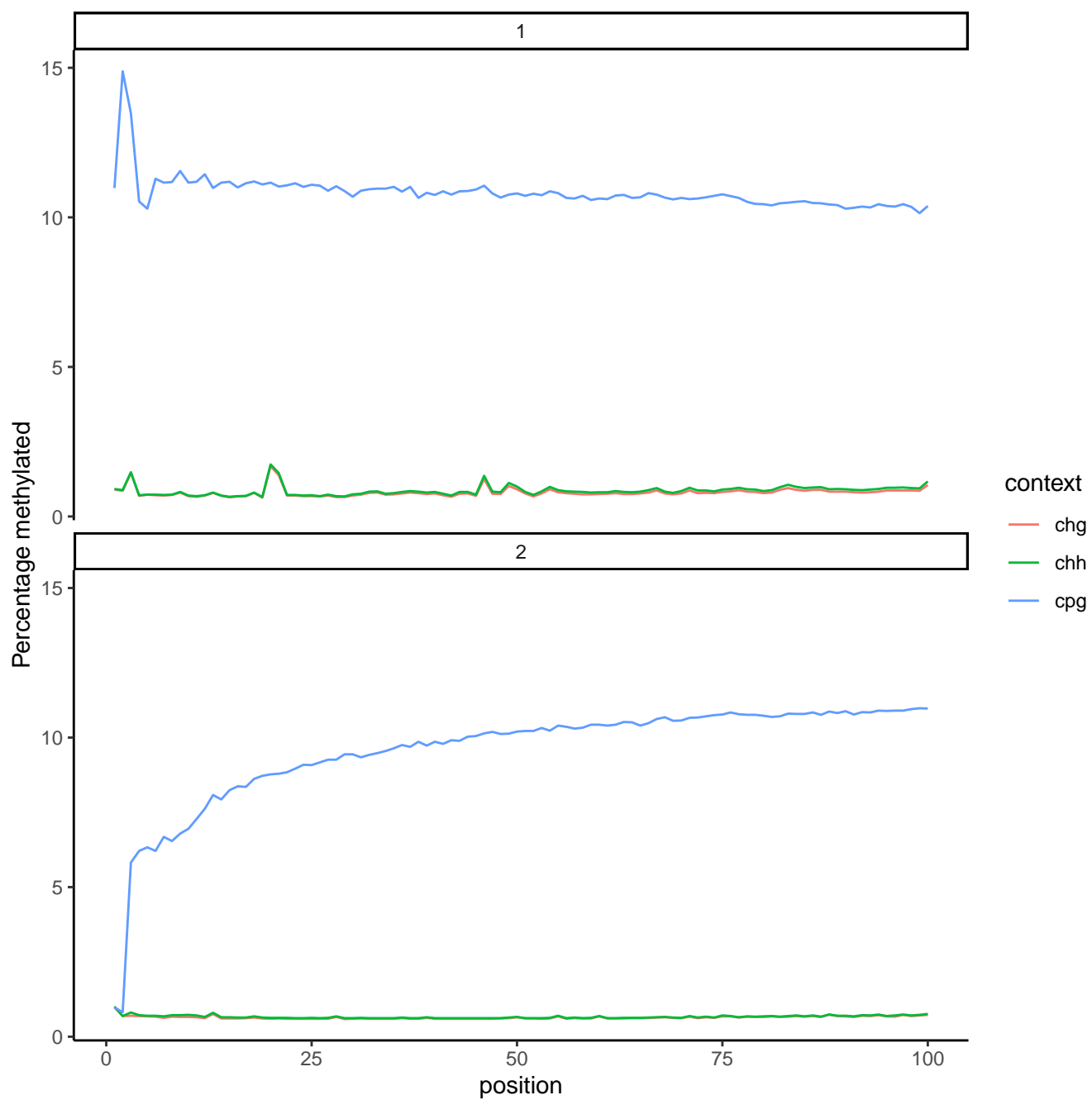

Supplement: Supplementary file 2 — Supplementary materials Figs S1–10 [file 41437_2024_724_MOESM2_ESM.zip › Spider Meth Figs S1-10 m-bias plots/Spider Meth Fig S5 T54 Alive.pdf]

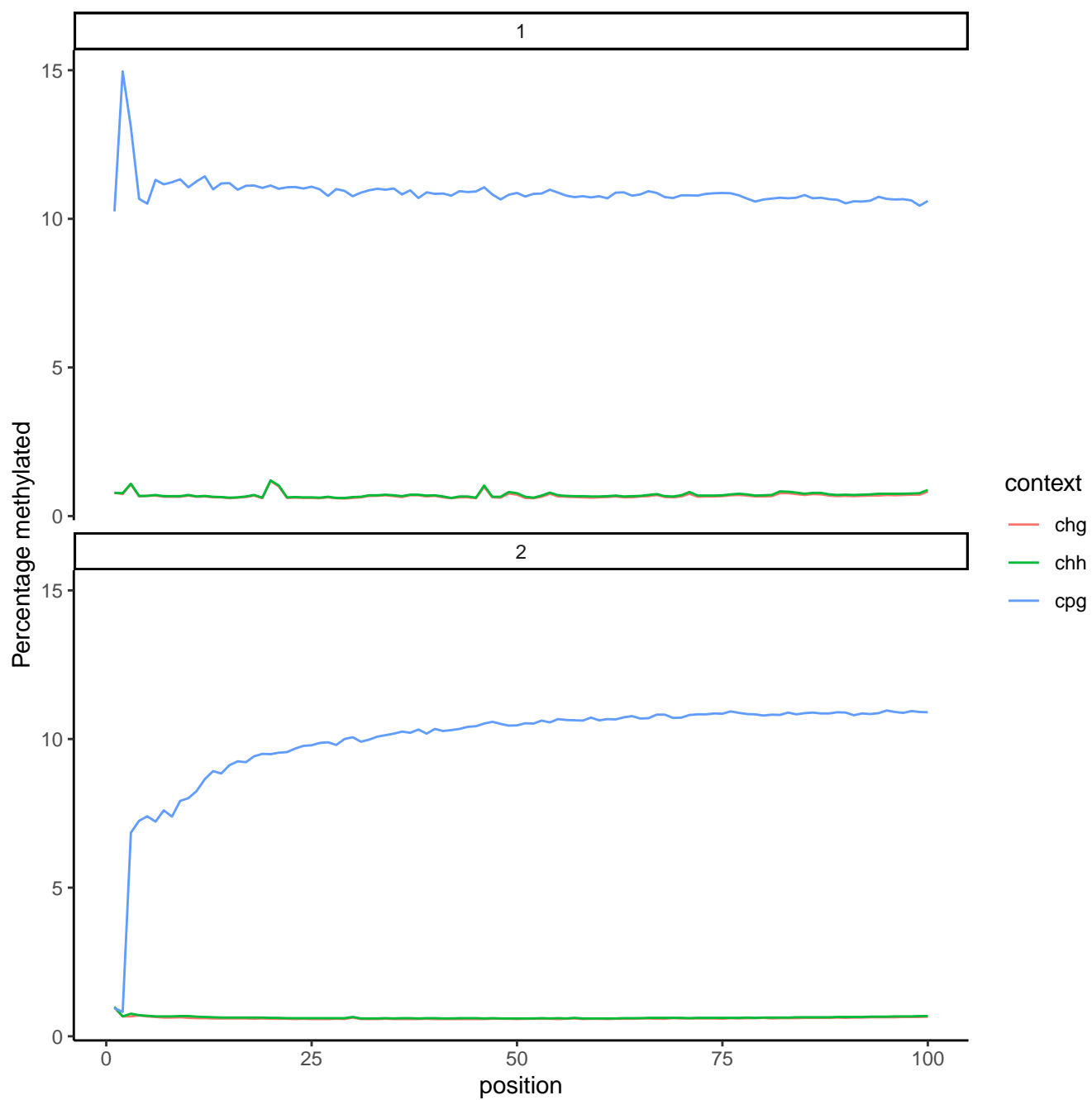

Supplement: Supplementary file 2 — Supplementary materials Figs S1–10 [file 41437_2024_724_MOESM2_ESM.zip › Spider Meth Figs S1-10 m-bias plots/Spider Meth Fig S6 T54 Dying.pdf]

1

15

10

5

0

Percentage methylated

context

chg

chh

cpg

2

15

10

5

0

0

25

50

75

100

position

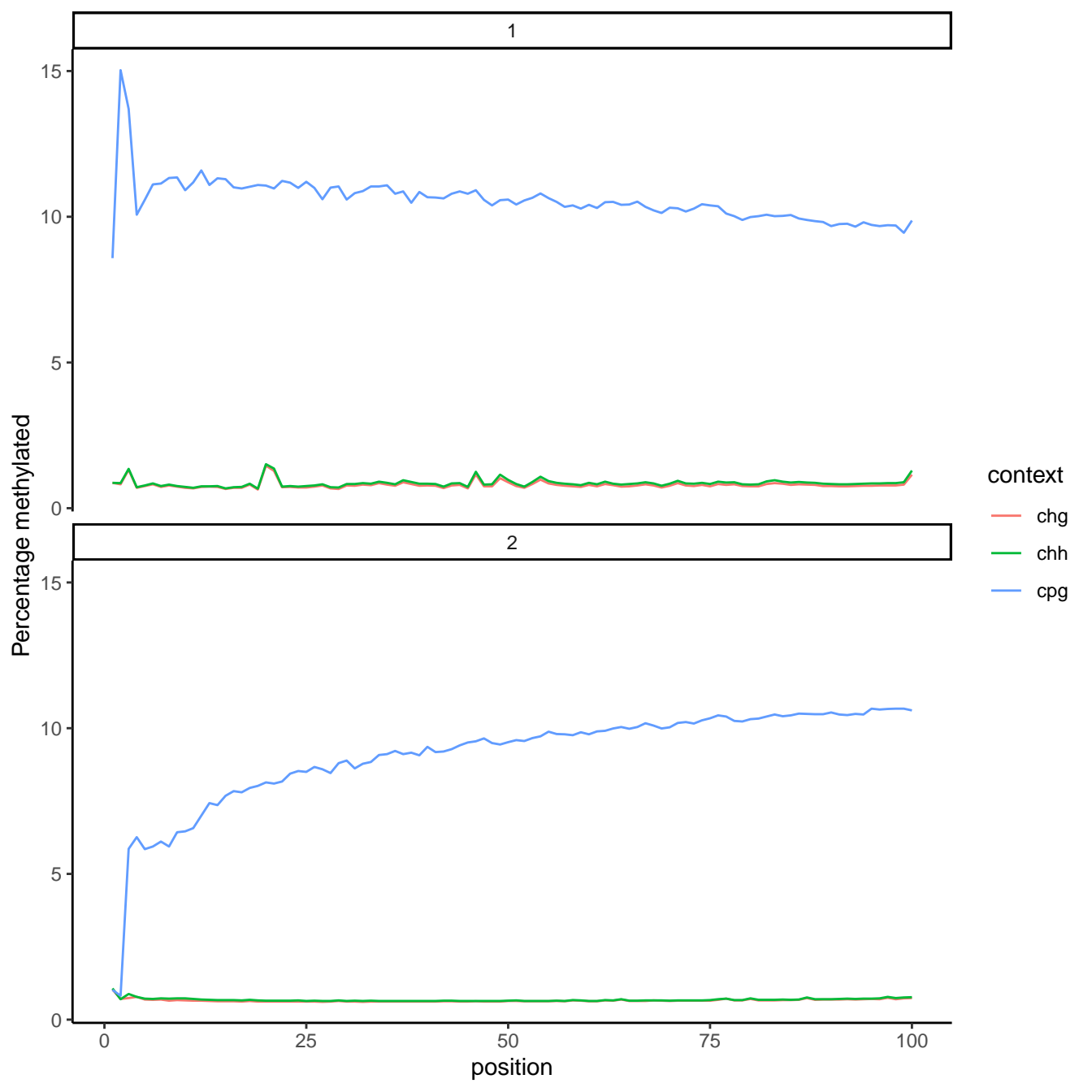

Supplement: Supplementary file 2 — Supplementary materials Figs S1–10 [file 41437_2024_724_MOESM2_ESM.zip › Spider Meth Figs S1-10 m-bias plots/Spider Meth Fig S7 T55 Alive.pdf]

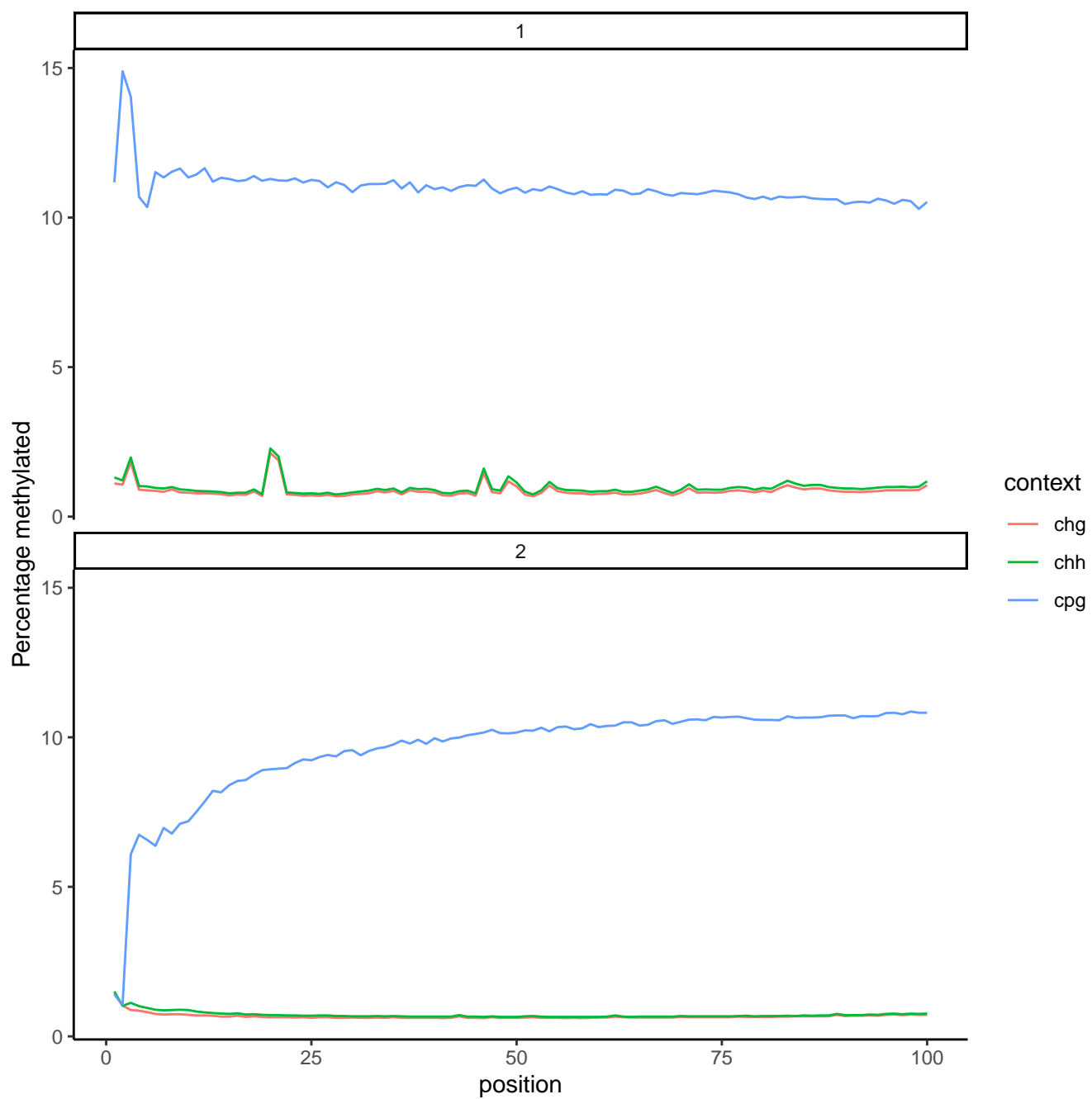

Supplement: Supplementary file 2 — Supplementary materials Figs S1–10 [file 41437_2024_724_MOESM2_ESM.zip › Spider Meth Figs S1-10 m-bias plots/Spider Meth Fig S8 T55 Dying.pdf]

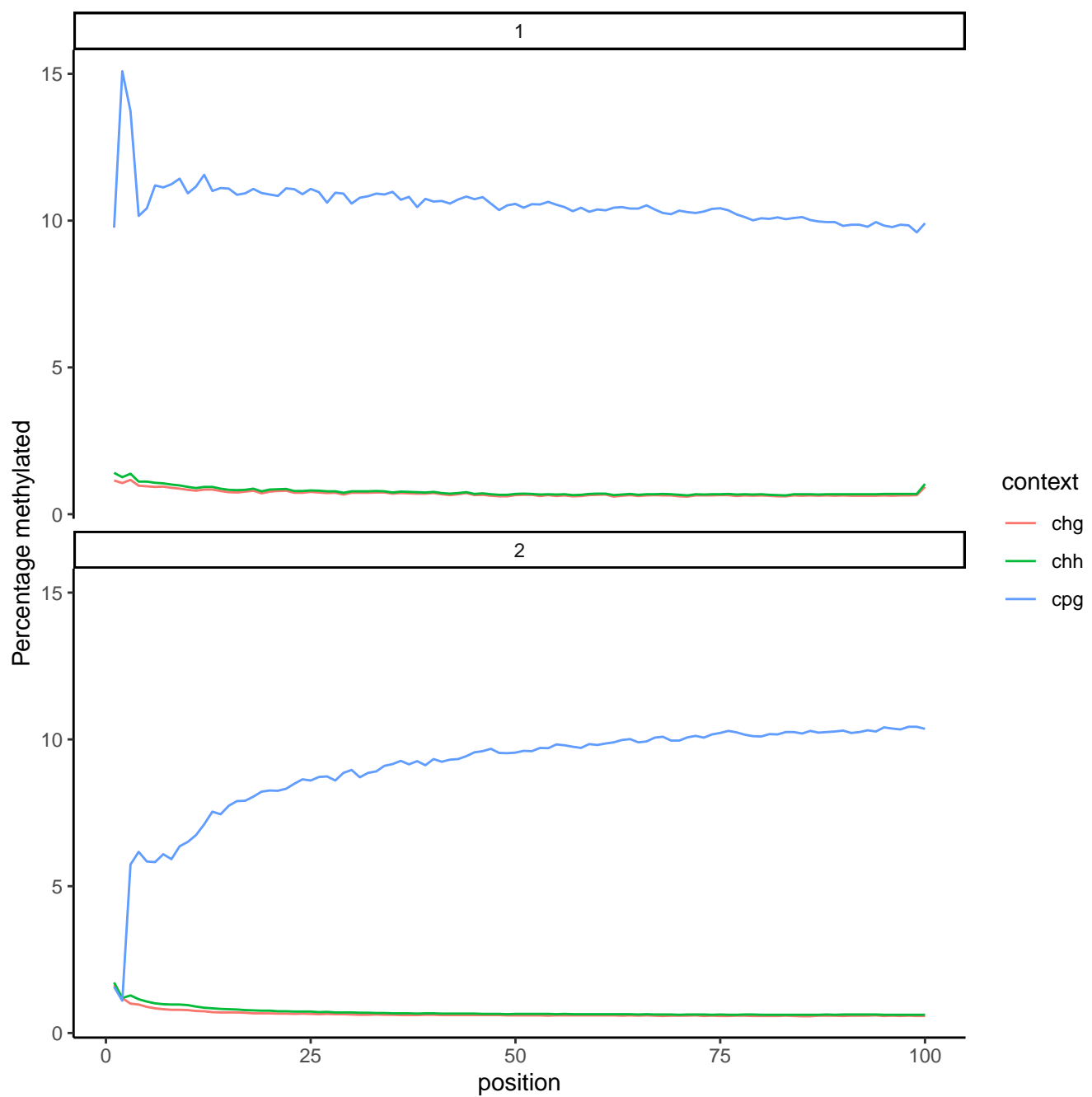

Supplement: Supplementary file 2 — Supplementary materials Figs S1–10 [file 41437_2024_724_MOESM2_ESM.zip › Spider Meth Figs S1-10 m-bias plots/Spider Meth Fig S9 T56 Alive.pdf]
